# Effect of Postbiotic Based on Lactic Acid Bacteria on Semen Quality and Health of Male Rabbits

**DOI:** 10.3390/ani11041007

**Published:** 2021-04-03

**Authors:** Jesús V. Díaz Cano, María-José Argente, María-Luz García

**Affiliations:** 1 Pentabiol S.L., Polígono Noain-Esquiroz s/n, 31110 Pamplona, Spain; jesus@pentabiol.es; 2 Centro de Investigación e Innovación Agroalimentaria y Agroambiental (CIAGRO-UMH), Miguel Hernández University, 03312 Orihuela, Spain; mj.argente@umh.es

**Keywords:** fermented food, hepatic profile, lactic acid bacteria, postbiotic, rabbit, semen profile

## Abstract

**Simple Summary:**

Postbiotics, especially those derived from metabolites of *Lactobacillus*, have been proposed as an alternative to the use of antibiotics for prevention and treatment of some diseases. This study was performed in rabbits due to their economic importance as a livestock species in Mediterranean countries, as well as being an experimental model in biomedicine. In this work, the use of a diet enriched with a postbiotic based on lactic acid bacteria is proposed to improve the seminal characteristics of rabbits and their health.

**Abstract:**

The aim of this study was to evaluate the effect of lactic acid bacteria-based postbiotic supplementation on semen characteristics and hematological and biochemical profiles in rabbits. A total of 28 males were randomly allocated into two groups. Males received a Control diet and Enriched diet supplemented with postbiotic for 15 weeks (4 weeks of adaptation period and 11 weeks of experimental period). Body weight, feed intake and semen characteristics were recorded weekly. Hematological profile was recorded at the beginning and end of the experiment and biochemical profile at 0, 5, 10 and 15 weeks. Bayesian methodology was used for the statistical analysis. Feed intake was higher in Control diet (125.2 g) than in the Enriched diet (118.6 g, *p* = 1.00). The percentages of abnormal spermatozoa were higher in Control diet than in Enriched diet (30% and 22%; *p* = 0.93) and the acrosome integrity percentage was lower (97% and 96%; *p* = 0.87). The hematological profile was within the range for healthy rabbits. The plasmatic level of alanine aminotransferase was higher in Control diet than Enriched diet at 5 and 10 weeks (*p* = 0.93 and *p* = 0.94, respectively) and alkaline phosphatase was similar in Control diet throughout the experiment, but decreased in Enriched diet (*p* = 0.97). No difference was found in kidney parameters (uric nitrogen and creatinine). Enriched diet showed higher total protein and globulin than Control diet (*p* = 0.99). Phosphorus was lower (*p* = 0.92) in Control diet than in Enriched diet. In conclusion, the addition of the postbiotic based on lactic acid bacteria seems to improve the quality of the semen and the liver profile in rabbits.

## 1. Introduction

Probiotics are live microorganisms which, when administered in adequate amounts, confer health benefits on the host [1]. Probiotic microorganisms are primarily lactic acid-producing bacteria of the genus *Lactobacillus* [2]. These probiotics can regulate the balance of gut microbes, promote the growth and productivity of animals and improve host resistance to diseases [3]. To this end, they have been extensively used in dairy cattle [4], beef cattle [5], pigs [6], hens [7] and rabbits [8]. Postbiotics are defined as soluble products or metabolites secreted by probiotics that have physiological benefits to the host [9]. Postbiotics consist of a wide range of effector molecules [10] and they are capable of reducing the gut pH and, in turn, inhibiting the proliferation of opportunistic pathogens in the feed and gut microbiota [10,11]. Postbiotics, especially those derived from metabolites of *Lactobacillus*, have been proposed as an alternative to the use of antibiotics, not only in humans, but also in monogastrics [12]. Currently, the application of postbiotics in human food, animal feed and pharmaceutical industries is increasing and postbiotic products derived from *Lactobacillus* species are commercially available for prevention or treatment of some diseases [10].

Rabbit is a livestock species reared either for the production of meat, hair or skin or as an experimental reference for other species, such as pigs or humans [13]. In rabbit meat production, artificial insemination is being widely used in intensive production farms [14]. The success of artificial insemination programs in rabbits depends to a great extent on both male health and reproductive performance [15]. Thus, the productivity, welfare and health of males should be improved by handling or feeding. Unlike other monogastric animals, data on the use of postbiotics in rabbits are quite scarce [12]. The aim of this study is to study the effect of supplementation with a postbiotic based on lactic acid bacteria on semen characteristics and hematological and biochemical profiles in male rabbits.

## 2. Materials and Methods

### 2.1. Ethics Statement

All experimental procedures were approved by the Miguel Hernández University of Elche Research Ethics Committee, according to Council Directives 98/58/EC and 2010/63/EU (reference number 2019/VSC/PEA/0163).

### 2.2. Product Description

The fermented food product tested was the result of a specific process of fermentation of a substrate and a combination of specific lactic acid bacteria and yeast. The substrate was a plant-based food product primarily composed of soya, alfalfa and wheat, along with other minor components. The fermented food product contained the phyla Firmicutes (38.7%), Proteobacteria (26.7%), Bacteroidetes (18.3%), Actinobacteria (14.5%) and Saccharibacteria (1.8%). At genus level, *Lactobacillus* was the predominant, accounting for more than 6% of identified species [16].

### 2.3. Animals

A total of 28 rabbit males aged between 9 and 12 months were used [17]. The animals were kept on an experimental farm at the Universidad Miguel Hernández de Elche (Spain). All animals were reared in individual cages (37.5 × 33 × 90 cm) during the entire experiment. The photoperiod was 16 h light:8 h dark.

### 2.4. Diets

Two diets were used. The control diet presented the following composition: 17% crude protein, 15% crude fiber, 9% crude ash, 3.6% crude fat, 1.2% calcium, 0.6% phosphorus and 0.3% sodium. The enriched diet presented the same composition supplemented with 2.0 kg of a fermented food product in a ton of feed.

### 2.5. Experimental Design

Animals were randomly divided into two groups of 14 males each; one group received the Control diet and the other the Enriched diet. Animals had a 4-week adaptation period to the feed. The experimental procedure lasted 11 weeks. Animal body weight and feed intake were recorded weekly.

### 2.6. Semen Collection and Evaluation

Two ejaculates per male were collected each week on a single day using an artificial vagina, with a minimum of 30 min between ejaculate collections. After the adaptation period, semen evaluations were performed for 11 weeks. If gel was present, it was removed. Only ejaculates exhibiting a white color were classified as normal and were evaluated. Ejaculates were diluted (dilution 1:5) with TRIS–citrate-glucose extender. Percentages of motile sperm were evaluated subjectively (from 0 to 5) under a microscope at a magnification of 400× with a thermostatic plate set at 37 °C.

An aliquot from each ejaculate (0.1 mL) was fixed with 0.9 mL of 2% glutaraldehyde solution in DPBS. The sperm concentration was determined using a Thoma-Zeiss cell counting chamber (Marienfield, Germany). A total of 100 spermatozoa were evaluated at a magnification of 400× with a differential interface contrast microscope (Normarski contrast). Spermatozoa were classified as normal or abnormal. The percentage of abnormal spermatozoa was calculated. Abnormalities were referred to tail, head and middle piece. Their percentages were calculated. Presence of cytoplasmic droplets and status of the acrosome (intact or damaged) in the normal spermatozoa were evaluated and their percentages were calculated.

### 2.7. Blood Collection and Biochemical and Haematological Parameters

Following the blood sampling procedure described in [18], blood samples were collected into a tube with tripotassium ethylenediaminetetraacetic acid (K3-EDTA) at weeks 0 and 15. Hematological parameters such as white blood leukocyte count (WBC, 103/μL) and percentage of lymphocytes, neutrophils, monocytes, basophils and eosinophils were determined with the Abacus Junior Vet hematology analyzer (Diatron, Austria).

Blood samples were collected into a lithium heparin tube at weeks 0, 5, 10 and 15. After centrifugation at 4000 rpm for 15 min, the concentrations of total bilirubin (TBIL, μmol/L), alkaline phosphatase (ALP, U/L), albumin (ALB, g/L), alanine aminotransferase (ALT, U/L), total protein (TP, g/L), globulin (GLOB, g/L), glucose (GLU, mmol/L), creatinine (CRE, μmol/L), uric nitrogen (BUN, mmol/L), amylase (AMY, U/L), calcium (Ca^2+^, mmol/L), potassium (K+, mmol/L), sodium (Na+, mmol/L) and phosphorus (FOS, mmol/L) were assessed. These biochemical parameters were determined with VETSCAN Comprehensive Diagnostic Profile Rotors (Diatron, Austria).

### 2.8. Statistical Analyses

#### 2.8.1. Survival, Body Weight and Feed Intake

A Kaplan–Meier plot was used for the survival analyses (GraphPad Prism 9.0.0)

Body weight and feed intake were analyzed using the following model:

Yijkl = µ + Wi + Dj + Wi × Dj + mijk + eijkl,

where Wi is the week effect (i = 15), Dj is the diet effect (j = 2; Control diet and Enriched diet); Wi × Dj is the interaction between week and diet, mijk is the random effect of the male and eijkl is the residual term. The body weight was also included as covariate for feed intake

#### 2.8.2. Seminal Parameters

The percentage of normal ejaculates was analyzed using Chi-square test. Seminal parameters were analyzed using the following model:

Yijklm = µ + Oi + Wj + Dk + mijkl + eijklm,

where Oi is the collection order effect (i = 2; first and second), Wj is the week effect (j = 11), Dk is the diet effect (k = 2; Control diet and Enriched diet), mijkl is the random effect of the male and eijklm is the residual term.

#### 2.8.3. Hematological and Biochemical Traits

Data were analyzed using the following model:

Yijkl = µ +Wi + Dj + Wi × Dj + mijk + eijkl,

where Wi is the week effect (i = 2, week 0 and 15 for haematological traits; i = 4, week 0, 5, 10 and 15 for biochemical traits), Dj is the diet effect (j = 2; Control diet and Enriched diet); Wi × Dj is the interaction effect, mijk is the random effect of the male and eijkl is the residual term.

Residuals and male effects were assumed to be independently normally distributed with the same variance. A Bayesian analysis was used, with bounded flat priors for all unknown parameters. Marginal posterior distributions were estimated for all unknowns using Gibbs sampling. Marginal posterior distributions of the differences between lines were computed with the Rabbit software program developed by the Institute for Animal Science and Technology (Valencia, Spain), using Monte Carlo Markov chains of 60,000 iterations, with a burn-in period of 10,000, and only 1 out of every 10 samples was saved for inferences. Convergence was tested using the Z criterion of Geweke and Monte Carlo sampling errors were computed using time-series procedures.

Results are presented with Bayesian methodology. We provide the difference between diets (D_D-E_) and the precision of our estimation, finding the shortest interval with 95% probability of containing the true value, which can be asymmetric around the estimation. This is called the highest posterior density interval at 95% probability. We also calculate the actual probability of the difference between the Control diet and Enriched diet |D_D-E_| being higher than zero. We consider that there is enough evidence for the Control and Enriched diets being different when the probability of this difference in absolute value |D_D-E_| is more than 90%.

## 3. Results

### 3.1. Survival, Body Weight and Feed Intake

**Figure 1:**
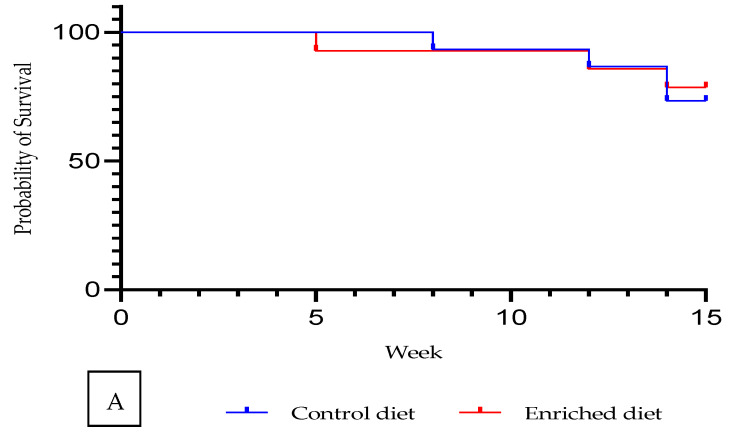

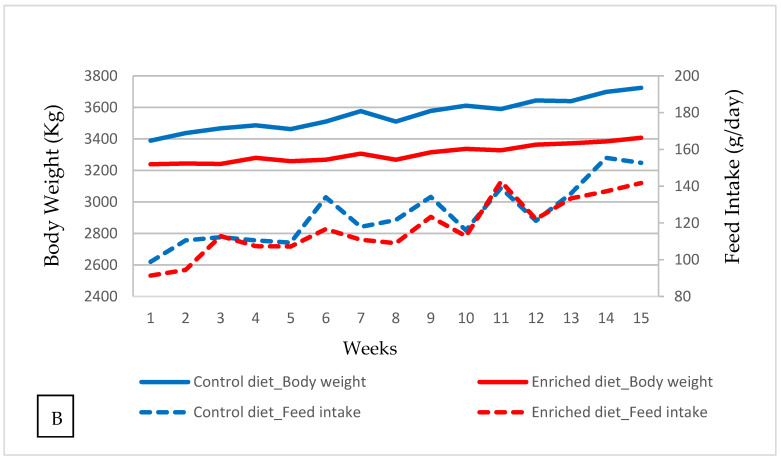
Control and Enriched diet: (**A**) Kaplan–Meier plot. (**B**) Evolution of body weight and feed intake.

Males fed with Enriched diet displayed a similar survival rate to those on Control diet (Figure 1a). Survival rate was 78.6% for Enriched diet and 73.3% for Control diet (Chi-square = 0.07; P value = 79%; data not shown in tables).

In general, body weight was 3514 g in Control diet and 3433 g in Enriched diet (*p* = 0.85, Table 1). Feed intake was 5% higher with the Control diet (125.2 g) than with the Enriched diet (118.6 g; *p* = 1.00). This difference was not due to a higher body weight of Control diet, as when the body weight was included as a covariate, the difference between diets was maintained. The evolution of the body weight and feed intake each week is shown in Figure 1b.

### 3.2. Sperm Quality

Both diets showed similar percentages of eliminated ejaculates due to low macroscopic quality (12% in the Control diet and 14% in the Enriched diet; Chi-square = 0.58; *p* = 45%; data not shown in tables).

Volume, motility and production were similar in both diets (Table 2). Enriched diet showed a lower percentage of abnormal spermatozoa than Control diet (22% and 30%, respectively; *p* = 0.93). This difference was due to the lower percentage of tail abnormalities (16% and 24%, respectively; *p* = 0.90). Similar percentages of head and middle piece abnormalities were found in both diets (4% and 2%, respectively).

A similar cytoplasmic droplet was shown for both diets (*p* = 0.69). Acrosome integrity was higher in Enriched than Control diet (97% and 96 % respectively; *p* = 0.87).

### 3.3. Hematological and Biochemical Parameters

**Figure 2:**
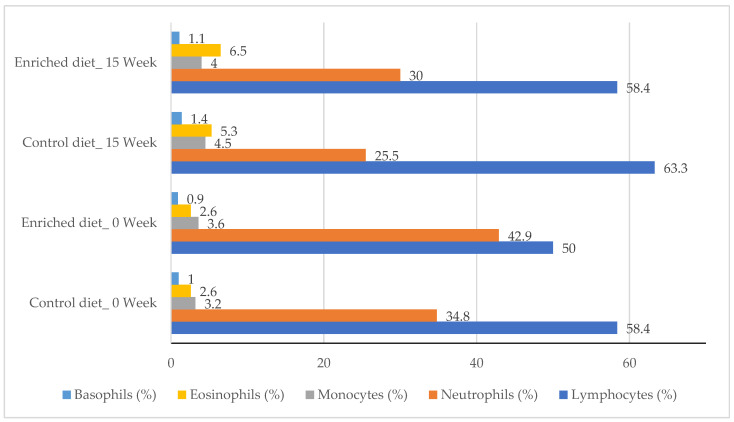
Percentage of lymphocytes, neutrophils, monocytes, eosinophils and basophils for Control and Enriched diets at 0 and 15 weeks.

Figure 2 shows the hematological parameters for diets at the beginning and end of the experiment. Lymphocytes increased by 15% and 20% in the Control diet (*p* = 0.90) and in the Enriched diet (*p* = 0.93). Monocytes increased for the Control diet (*p* = 0.97), but they did not vary in the Enriched diet. Neutrophils decreased in the Control diet (*p* = 0.90) and in the Enriched diet (*p* = 0.99). Eosinophils and basophils increased from week 0 to 15 in both Diets (*p* = 1.00 and *p* = 0.91, respectively). WBC did not vary between diets or throughout the experiment, ranging between 8.4 and 9.6 × 10^3^/μL (data not shown in tables).

**Figure 3:**
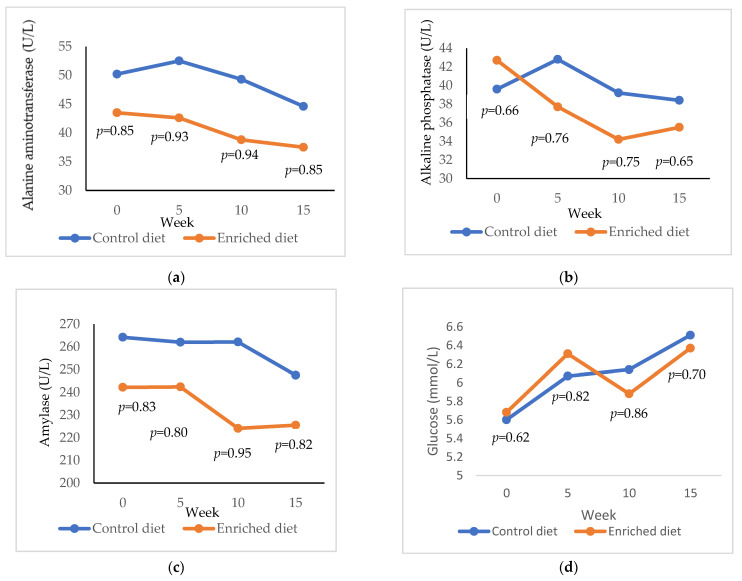
Evolution of (**a**) alanine aminotransferase; (**b**) alkaline phosphatase; (**c**) amylase; (**d**) glucose in males fed with Control and Enriched diet. *p* is the probability of the difference being >0 when the difference between the diets was >0 or being <0 when this difference was <0.

Alanine aminotransferase is shown for Control and Enriched diets at 0, 5, 10 and 15 weeks in Figure 3a. Alanine aminotransferase was higher in the Control diet than in the Enriched diet at 5 weeks (*p* = 0.93) and at 10 weeks (*p* = 0.94). Both diets decreased the levels of alanine aminotransferase, but this decrease was lower in Control diet (5.6 U/L; from 50.2 to 44.6 U/L) than in Enriched diet (6.0 U/L; from 43.5 to 37.5 U/L; *p* = 0.95; results not shown in Figure). Alkaline phosphatase was similar for both diets and throughout the entire control period (Figure 3b). Nevertheless, while the difference between 0 and 15 weeks was similar in Control diet (39.6 and 35.5 U/L, respectively; *p* = 0.62), the alkaline phosphatase exhibited relevant reduction in Enriched diet (42.7 and 35.5 U/L, respectively; *p* = 0.97). Amylase tends to be higher in Control diet than in Enriched diet, showing differences at week 10 (*p* = 0.95; Figure 3c). Glucose was similar for both diets and ranged from 5.6 to 6.5 mmol/L (Figure 3d).

**Figure 4:**
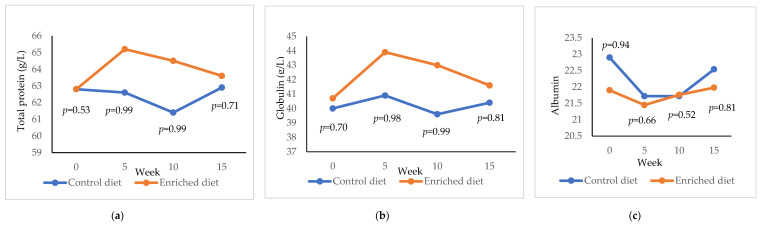
Evolution of (**a**) total protein; (**b**) globulin; (**c**) albumin in males fed with Control and Enriched diet. *p* is the probability of the difference being >0 when the difference between the diets was >0 or being <0 when this difference was <0.

Enriched diet showed a higher total protein than Control diet after the adaptation period (+ 2.68 g/L; *p* = 0.99; Figure 4a) and was maintained until week 10 (+3.09 g/L; *p* = 0.99). However, after feeding Enriched diet for 15 weeks, the total protein was similar to Control diet. Control diet showed a lower globulin concentration than the Enriched diet at both 5 (*p* = 0.98; Figure 4b) and 10 weeks (*p* = 0.99). Albumin was higher at the start of the experiment in the Control diet (22.9 g/L; Figure 4c) than in the Enriched diet (21.9 g/L; *p* = 0.94). Both diets presented similar albumin from 5 to 15 weeks.

**Figure 5:**
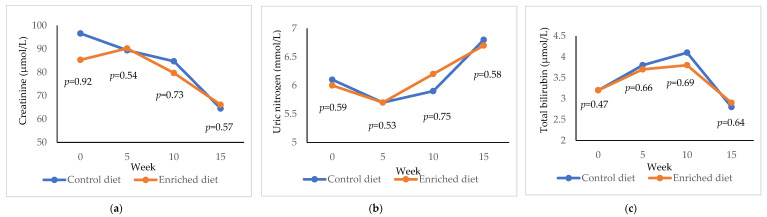
Evolution of (**a**) creatinine; (**b**) uric nitrogen; (**c**) total bilirubin in males fed with Control and Enriched diet. *p* is the probability of the difference being >0 when the difference between the diets was >0 or being <0 when this difference was <0.

Control diet showed higher creatinine values than the Enriched diet (*p* = 0.92) at week 0, but the values were similar at weeks 5, 10 and 15 (Figure 5a). Both diets decreased creatinine during the experiment (−20.8 µmol/L in Control diet, *p* = 1.00; −30.5 µmol/L in Enriched diet, *p* = 1.00). Regarding uric nitrogen, a similar concentration was shown for both diets (Figure 5b) and uric nitrogen increased during the experiment (+0.7 mmol/L in both lines; *p* = 0.99). Total bilirubin was similar in both diets (Figure 5c) and decreased during the experiment (−0.3 µmol/L in Control diet, *p* = 0.92; −0.4 µmol/L in Enriched diet, *p* = 0.96).

**Figure 6:**
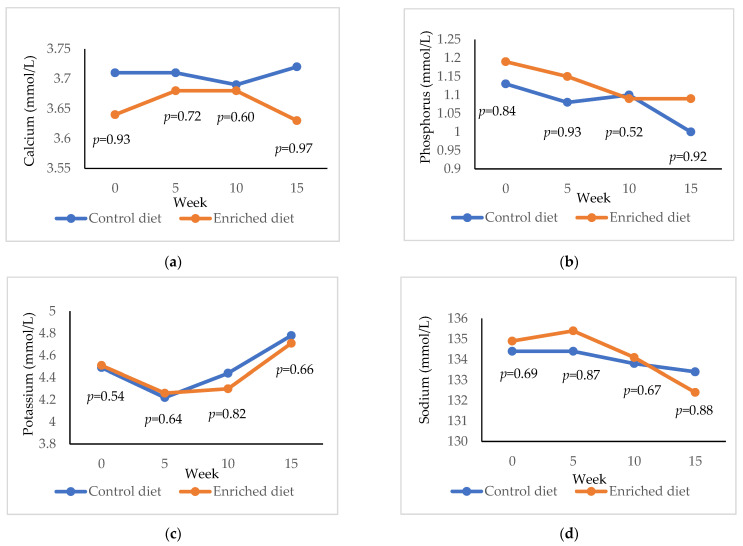
Evolution of (**a**) calcium; (**b**) phosphorous; (**c**) potassium; (**d**) sodium in males fed with Control and Enriched diet. *p* is the probability of the difference being >0 when the difference between the diets was >0 or being <0 when this difference was <0.

The results for calcium, phosphorus, potassium and sodium are presented in Figure 6. Calcium was higher in Control diet both at 0 weeks (*p* = 0.93) and at 15 weeks (*p* = 0.97) and phosphorus was lower at 4 weeks (*p* = 0.90) and 15 weeks (*p* = 0.92). Potassium and sodium were similar for the two diets throughout the experimentation period.

## 4. Discussion

There is increasing evidence of the role of postbiotics as health promoter. The beneficial effects of postbiotics are mediated through an interaction between the microbial products and the host [10]. In this study, we tested the effectiveness of a postbiotic formulated with a fermented food product in terms of semen quality and health status of male rabbits. Postbiotics have recently demonstrated the ability to improve welfare and health in diabetic rats [16] and dairy heifer calves [19,20].

Food intake was lower with the postbiotic than with control diet from the second week. Nevertheless, survival was not affected. When this diet has been applied to dairy heifer calves, there was also a decrease in consumption from week 5 of intake [19].

Many studies have been carried out to improve the seminal quality in rabbits by supplementing feed with probiotics [21,22]. To the best of our knowledge, no information has been found regarding postbiotics. In our experiment, a slight improvement in the acrosome integrity and spermatozoa with normal tail was obtained in the Enriched diet, although an increase in motility was not achieved.

Hematological parameters provide valuable information on the health status of the animal. In the present study, the hematological profiles were within the range for healthy rabbits both at the beginning and end of the experiment and for both diets [18,23]. Levels of albumin, alkaline phosphatase, alanine aminotransferase, total bilirubin, total protein, globulin, glucose, creatinine, uric nitrogen and amylase are within the wide range of values reported in rabbits [18,24,25].

Alanine aminotransferase and alkaline phosphatase are markers of hepatic diseases [26,27] and alkaline phosphatase is also related to other disorders such as increased bone deposits, intestinal damage, hyperthyroidism and generalized tissue damage [28]. Males fed with postbiotic diet showed lower alanine aminotransferase and alkaline phosphatase concentration, thus the liver profile was improving. The benefit of the postbiotic on liver function has also been demonstrated in rats [16]. Alanine aminotransferase decreased in meat rabbits fed with lactic acid bacteria additives [29]. Moreover, a negative correlation between these biomarkers in plasma and semen quality, mainly the motility and the acrosomal damage, has been reported in rabbits [30] and in goats [31]. As previously mentioned, the improvement in acrosome and tail would agree with this result.

Elevated glucose levels are generally due to various stress factors [32]. Several studies have reported the hypoglycemic effect of probiotic and fermented products [33,34]. Our result indicates that amylase tends to be lower with the postbiotic. This effect is not immediate, but it occurs after consuming the diet for 10 weeks. Although the glucose levels were not modified in this study, they were attenuated with the fermented food product in rats due to changes in the gut microbiota composition [16].

Principal plasma proteins are albumin and globulin [35]. Globulin can be considered as a good indicator of immunity response [36]. The fermented product increased total protein by 2.5% and globulin by 5.2%, whereas the albumin concentration was similar in both diets. Thus, it could be indicated that postbiotic improves immunity to infectious agents. Similar results have been obtained in calves supplemented with this postbiotic [21]. It has been found that postbiotics from *Lactobacillus plantarum* also confer anti-inflammatory responses, as observed in a study in porcine intestinal epithelial cell lines [37]. Regarding seminal quality, higher levels of albumin decrease sperm abnormality and increase acrosomal integrity, whereas these parameters are not affected by total protein and globulin [38]. So, the increase in globulins and total proteins does not seem to have a direct effect on the improvement of sperm quality.

We measured uric nitrogen and creatinine as biomarkers of kidney function status. The results indicate that kidney function was not affected by the use of the postbiotic, as both biomarkers evolved in a similar way during the experiment for the Control and Enriched diet. Uric nitrogen could serve as substrate for reactive oxygen species and thus protect important biomolecules against oxidative damage of the spermatozoa [39]. Nevertheless, no significant changes in concentration of uric nitrogen either in semen plasma or blood are provided with supplemented diets [40].

Little information is available on supplementation of blood minerals in response to postbiotics. Minerals act as structural and functional cofactors in metal-containing enzymes [41]. In addition, phosphorus is part of the ATP molecule, which is the major energy source for cellular function [42]. The postbiotic increased phosphorous levels in rabbit blood. This finding is supported in rabbits fed with probiotics and an improvement in the metabolic state of the rabbits could be expected [41]. It is well known that increased concentrations of phosphorous are associated with increased fertility of males [40]. Nevertheless, the results regarding calcium are not conclusive. The postbiotic equalizes the calcium levels of the animals with those of the Control diet, although the calcium decreased to the initial values in the last week of treatment.

## 5. Conclusions

In conclusion, postbiotics based on lactic acid bacteria improve the health status of rabbit males, especially with respect to the liver function. Sperm quality was also improved, specifically the quality of the tail and acrosome of the spermatozoid. The improvement in postbiotic intake should be investigated, as it could affect results obtained in the long term.

## Figures and Tables

**Table 1:** Effect of diet on body weight and feed intake in male rabbits.

	D	E	D_D-E_	HPD_95%_	P
Body weight (g)	3514	3443	71	−66, 202	0.85
Feed intake (g/day)	125.2	118.6	6.6	2.0, 10.7	1.00
Feed intake (g/day) *	125.3	118.3	7.0	2.7, 11.4	1.00

D: Median of the Control diet; E: Median of the Enriched diet; D_D-E_: Difference between the Control and Enriched diet; HPD_95%_: Highest posterior density region at 95%; P: Probability of the difference being >0. * Body weight as covariate.

**Table 2:** Effect of diet on sperm quality in male rabbits.

	D	E	D_D-E_	HPD_95%_	P
Volume (mL)	1.09	1.13	0.04	−0.27, 0.18	0.64
Motility	3.72	3.75	−0.03	−0.07, 0.62	0.53
Production (10^6^ spz)	266.2	269.1	−3.3	−75,7, 63.1	0.54
Abnormal spz (%)					
Total (%)	30	22	8	−2, 18	0.93
Head (%)	4	4	0	−3, 2	0.64
Tail (%)	24	16	8	−5, 18	0.90
Middle piece (%)	2	2	0	−1, 1	0.62
Cytoplasmic droplet (%)	12	10	2	−5, 8	0.69
Acrosome integrity (%)	96	97	−1	−3, 1	0.87

D: Median of the Control diet; E: Median of the Enriched diet; D_D-E_: Difference between Control and Enriched diet; HPD_95%_: Highest posterior density region at 95%; P: Probability of the difference being >0 when D_D-E_ > 0 or being <0 when D_D-E_ < 0.

## Data Availability

The data presented in this study are available on request from the corresponding author.
